# Measuring Chemical LLM robustness to molecular representations: a SMILES variation-based framework

**DOI:** 10.1186/s13321-025-01079-0

**Published:** 2025-10-30

**Authors:** Veronika Ganeeva, Kuzma Khrabrov, Artur Kadurin, Elena Tutubalina

**Affiliations:** 1 AIRI, 6 Presnenskaya, Moscow, Russia 123112; 2 National Research University Higher School of Economics (HSE University), 11 Pokrovksy Bulvar, Moscow, Russia 109028; 3 ISP RAS Research Center for Trusted Artificial Intelligence, Moscow, Russia; 4 Sber AI, Kutuzovsky Prospekt, 32, 121170 Moscow, Russia

**Keywords:** LLMs, Augmentations, SMILES, Molecule description task, Molecular properties regression and classification

## Abstract

The recent integration of natural language processing into chemistry has advanced drug discovery. Molecule representations in language models (LMs) are crucial to enhance chemical understanding. We explored the ability of models to match the same chemical structures despite their different representations. Recognizing the same substance in different representations is an important component of emulating the understanding of how chemistry works. We propose Augmented Molecular Retrieval (AMORE), a flexible zero-shot framework for the assessment of chemistry LMs of different natures. The framework is based on SMILES augmentations that maintain a foundational chemical structure. The proposed method facilitates the similarity between the embedding representations of the molecule, its SMILES variation, and that of another molecule. Experiments indicate that the tested ChemLLMs are still not robust to different SMILES representations. We evaluated the models on various tasks, including the molecular captioning on ChEBI-20 benchmark and classification and regression tasks of MoleculeNet benchmark. We show that the results’ change after SMILES strings variations align with the proposed AMORE framework.

## Scientific contribution

We present AMORE, a framework for evaluating chemical language models (ChemLMs) based on their inner embedding space. AMORE uses augmentations that reformulate SMILES strings of molecule structures to assess chemical representations. The proposed framework allows evaluation of ChemLMs without expensive manually annotated data.

## Introduction

Drawing inspiration from the Transformer-like architectures commonly used in NLP [1], the pharmaceutical community has embraced new, state-of-the-art molecule generation methodologies. This includes leveraging LM-based approaches such as ChemBERTa, T5Chem, ChemFormer, and BARTSmiles [2–5]. In particular, SMILES (Simplified Molecular Input Line Entry System) [6] is a commonly employed molecular representation type which enables researchers to supply language models with molecules in a string-based format. Single-domain models like those mentioned above are usually pre-trained on large SMILES datasets like ZINC-15 [7], then fine-tuned for downstream tasks like reaction modeling and chemical property prediction on datasets like USPTO [8, 9] and MoleculeNet [10].

Recently, LMs like MolT5 [11], Text+Chem T5 [12] and nach0 [13] have been introduced to integrate chemical and linguistic knowledge. These models were pre-trained on both chemical notations and natural text data, e.g., the large C4 [14] and ZINC-15, and fine-tuned on cross-domain tasks like molecule captioning.

However, the evaluation of downstream tasks does not directly assess the underlying chemical knowledge. One way of evaluating how aware the model is of the chemical principles is by checking if it can recognize slightly different string-based representations as describing the same chemical substances.

**Fig. 1:**
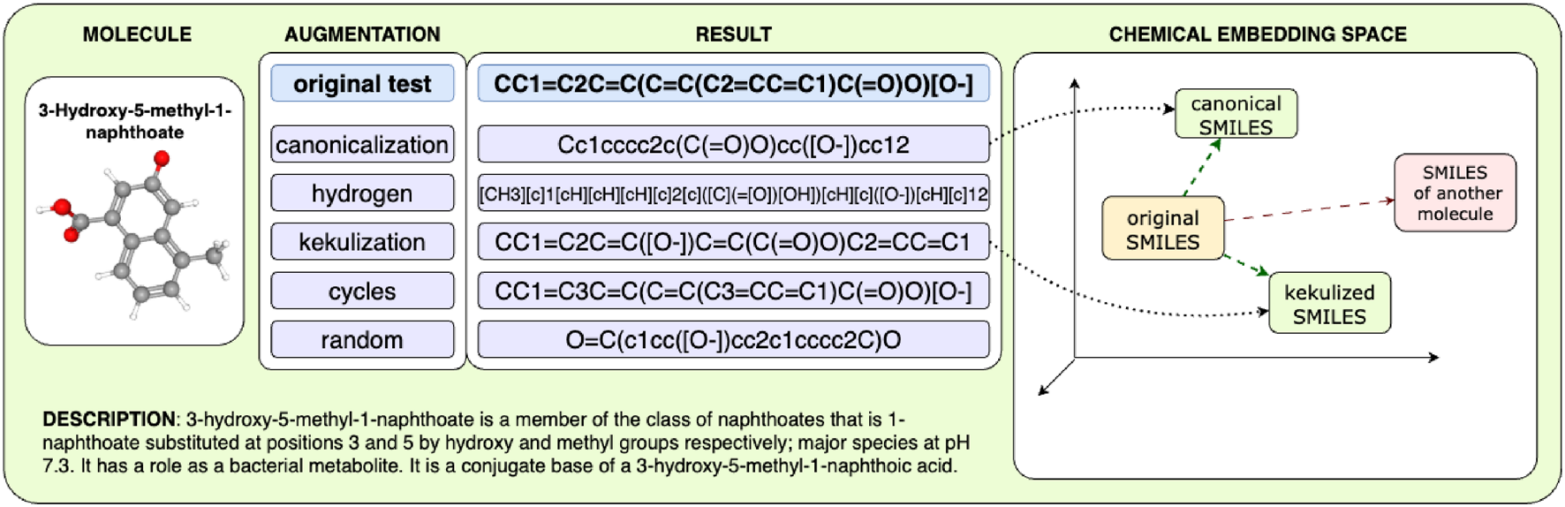
Our evaluation technique involves generating augmented representations for all molecules in a dataset using one of four augmentation procedures. After encoding original molecules and augmented SMILES representations and calculating distances between their embeddings, the study determines model performance based on top-1 accuracy, where the correct augmented SMILES must be retrieved at the top rank: that would mean that, according to the model’s knowledge, the augmented string encodes the same chemical as the original one. Conversely, the lower the correct SMILES ranks, the more pseudo-semantic distance the model puts between two strings encoding the same chemical, and so the less it is aware of the difference between the rules governing the chemical Signified and the textual Signifier

In this paper, we examine the understudied research question (RQ): *Do evaluation metrics used on chemical language models (ChemLMs) represent information about the level of their chemical knowledge, or do the models simply imitate chemical understanding by learning textual features?* Getting this answer is critical when it comes to such delicate fields as pharmaceutics and, more broadly, healthcare, where missteps in judgement translate into consequences more serious than skewed data. We also introduce the novel unified approach (Fig. 1) to verify if ChemLMs have effectively grasped the fundamental rules on the construction of molecular representations, such as SMILES. Our hypothesis posits that augmentation (creating various valid representations of the same molecule) should not significantly alter the similarity score between distributed representations of molecules and their augmented versions. To address the RQ, we conduct experiments using BERT-based [15], GPT-based [16], and T5-based [14] models. Our work is designed to provide insights into the extent to which ChemLMs can discern molecular structures and the impact of augmentation on their performance. To summarize, our main contributions are as follows:

- We show that despite ChemLMs failing to provide factual information about molecule features and precursors in generated descriptions, the state-of-the-art metrics can not fully reflect it.
- Extensive evaluation has revealed that embedding space of the existing state-of-the-art uni-modal and cross-modal chemical LMs are not robust to four SMILES augmentation types known to be identity transformations in terms of the underlying molecules.
- We propose **A**ugmented **Mo**lecular **Re**trieval (AMORE),^1^ a novel framework for quality assessment of chemical language models. It relies on augmentations of molecular SMILES string representations that are known to produce alternative representations without changing an underlying molecule. Unlike supervised fine-tuning-based methods for chemical LM quality assessment adopted from NLP, our framework adopts known chemical facts to perform a fully unsupervised evaluation.

In comparison to the conference version [17], we have: (1) examined the performance of the recently released nach0 model on augmentations and our framework, providing new experimental results and conclusions; (2) significantly enhanced the qualitative analysis of model inference; (3) conducted an error analysis and addressed the limitations of our framework; and (4) explored factors that may lead to chemically critical errors in the outputs during the molecule captioning task.

We begin Sect. 2 by discussing various approaches to evaluate generative language models in chemistry. We also offer a chemical and linguistic perspective on the augmentation of textual molecule representations.

In Sect. 3, we introduce the AMORE framework, which aids in exploring molecule representation embeddings and interpreting the model’s textual input. Section 4 presents our research results, including detailed evaluations with state-of-the-art metrics on augmented textual representations and comparisons with AMORE and METEOR. Section 5 discusses the results, followed by conclusions in Sect. 6.

## Background

***Molecule representations***

Chemical language models (ChemLMs) rely on text-based formats to represent molecules. Among several available formats–like InChI and SELFIES–we focus on SMILES (Simplified Molecular Input Line Entry System), a widely adopted method that encodes molecules as short ASCII strings. In SMILES format, for example:

- Atoms are represented with symbols (C for carbon, O for oxygen).
- Bonds are implied or denoted with symbols like = for double bonds.
- Branches use parentheses, and rings are indicated by matching numbers.

A single molecule can have multiple valid SMILES representations, depending on:

- The starting atom (atom order)
- How branches are arranged
- How rings are labeled
- Whether hydrogens are included explicitly
- Stereochemistry (3D configuration)

These differences permit the same molecule to be written in different–but chemically equivalent–ways. This flexibility is powerful, but it also introduces ambiguity for machine learning models. When we “augment” a SMILES string, we rewrite it using valid alternative formats without changing the underlying molecule. These augmented versions are like synonyms in natural language. A robust chemical model should treat these different representations as ones that mean the same thing.

***Chemical Syntax vs. Language Syntax***

The differences between SMILES strings can be compared to how natural language rearranges words without changing meaning: “Language models are used for biomedical and chemical tasks” vs. “Language models are used for chemical and biomedical tasks” Both are grammatically correct and convey the same message. We expect that a ChemLM trained on large SMILES datasets should understand that augmented SMILES are equivalent. If it fails to do so, this may indicate that the model is overfitting to specific string patterns rather than learning chemistry. Similarly, SMILES augmentations are comparable to the classic linguistic example:

- *Colorless green ideas sleep furiously*
- *Green colorless ideas sleep furiously*
- *Red colorless ideas sleep furiously*

The first two have similar grammar and meaning. The third, though similar in structure, has a different semantic sense–just like a SMILES string representing an entirely different molecule.

***Why Standard NLP Metrics Fall Short in Chemistry***

Most models are evaluated using text-based metrics from NLP, like BLEU (Bilingual Evaluation Understudy, [18]), ROUGE [19], and METEOR [20].

While useful for comparing word overlap or sentence fluency, these metrics have major limitations in chemistry:

- They emphasize exact word matching, which ignores deeper chemical meaning.
- They penalize valid but differently phrased captions.
- They cannot detect critical structural changes (e.g., a double bond becoming a single bond).

Modern embedding-based metrics like BERTScore or BLEURT also fall short. These models were trained on general text (like Wikipedia), not chemistry. So, small structural changes in a molecule may go unnoticed, or two valid representations of the same molecule may appear unrelated.

***Why we propose AMORE***

To address these shortcomings, we introduce AMORE, a method that compares molecular embeddings directly rather than relying on textual similarity. AMORE measures how stable a model’s internal representation is across different SMILES variants–something no NLP metric can do reliably. By focusing on chemical identity rather than word overlap, AMORE gives a more accurate view of whether models truly “understand” molecules or just memorize text patterns.

## AMORE: augmented molecular retrieval

In this section, we introduce AMORE, a flexible embedding-based evaluation framework for Language Model analysis in the chemistry domain.

***Concept***

We developed a method specifically designed to test chemical language models by leveraging the principle that synonymous molecular representations—despite differing textual encodings—should produce similar or identical embeddings. This assumption is grounded in the idea that such representations encode the same semantic entity: a single molecule. In natural language processing, similar approaches have been used to access linguistic understanding, where synonyms are expected to yield semantically aligned embeddings [1–3]. In chemistry, variations of the same molecule (e.g., different SMILES notations) are not only stylistic but structurally equivalent, making them ideal for probing whether models capture true molecular semantics.

Our framework is based on three core components: (1) SMILES augmentation, (2) embedding distance analysis, and (3) ranking of nearest neighbors. First, we generate multiple SMILES variants for each molecule, ensuring that they represent the same chemical structure through permutations like randomized atom orderings or aromaticity conventions. Second, we compute distances (e.g., Euclidean or cosine similarity) between embeddings of these variants to assess consistency. Finally, we evaluate how many embeddings of the same molecule are out-ranked by those of other molecules, quantifying the model’s ability to preserve molecular relationships. By combining these elements, our method provides a targeted evaluation of whether chemical models learn invariant representations, offering insights into their robustness and semantic fidelity.

***Methodology***

Our evaluation metrics are built on distributed representations of molecules and their augmentations.

Let $X_1$ denote the dataset comprising original representations of molecules, represented as $x_1, x_2, \ldots , x_n$. Through SMILES augmentation, we generate the $X_1'$ dataset, containing augmented representations of the same molecules, represented as $x_1', x_2', \ldots , x_n'$. In each experiment, a model encodes the augmented SMILES representations of molecules. Let $e(x_i)$ represent the embedding of SMILES $x_i$ from the original dataset, and $e(x_j')$ represent the embedding of the augmented SMILES $x_j'$ from the augmented dataset, where $i, j$ denote indices corresponding to molecules. The distance between embeddings $e(x_i)$ and $e(x_j')$ is calculated using a distance metric such as Euclidean distance. Suppose the nearest embedding from the augmented dataset is not an augmentation of the original SMILES embedding, i.e., $j \ne i$. In that case, it is inferred that the model does not recognize the same chemical structure in augmented textual representations. In other words, we use the top-*k* accuracy as the evaluation metric: $\mathrm {Acc@k}$ if the correct augmented SMILES is retrieved at the rank $\le k$, otherwise $\mathrm {Acc@k}=0$; in our case, *k*=1. In addition, we compute the Mean Reciprocal Rank (MRR) metric. This ranking metric can get a better sense of the performance degradation with the augmented SMILES strings since it reflects the average ranking of true molecule [21].

The practical objective of our approach is to compare embeddings for different textual representations of the same molecule structure. We use the fast nearest neighbor search library FAISS [22] that is efficient in a large-scale setting. Our methodology’s theoretical implications lie in understanding how efficiently ChemLMs reconstruct molecule structures from the textual representations provided to them.

***Augmentation Procedures***

We follow four popular augmentations from [23], where the authors showed that augmentations led to a decrease in ROUGE scores [19] in the molecule captioning task when evaluated two cross-domain T5 models, Text+Chem T5 and MolT5. Additionally, we add random atom order augmentation. We adopt the following SMILES-based augmentation procedures:

1. **Canonicalization**: we transform SMILES strings into a standardized RDKit string [24, 25], reducing ambiguity and facilitating accurate molecule comparisons.
2. **Hydrogen**: the presence of hydrogen atoms can significantly impact molecular properties and reactions [26]. In SMILES representations, hydrogen atoms are typically omitted, as their positions can be inferred based on standard valency rules. While the restoration of implicit hydrogens is trivial at the molecular graph level, explicitly adding hydrogens significantly alters the structure of the SMILES string. This augmentation introduces greater syntactic complexity, which can challenge language models by increasing the variability and depth of the SMILES grammar.
3. **Kekulization**: Aromaticity is an essential concept in organic chemistry, influencing molecular stability, reactivity, and spectroscopic properties. This involves transforming a SMILES string into a Kekulized SMILES string, where the aromatic $\pi$-electrons are static between every second carbon;
4. **Cycles**: In chemical graph theory, cycles (or rings) play a fundamental role in characterizing molecular structure and properties. Valid replacement of cycle numerical identifiers with other random numbers allows for testing the robustness of models in recognizing cyclic structures and their connectivity, providing insights into their ability to handle diverse molecular topologies.
5. **Random**: In contrast to the canonical SMILES generation algorithm, where the atom traversal order is deterministic, in this case it is randomized.

The key property of the five augmentations listed above is that the resulting augmented SMILES represents the same molecule as the original non-augmented one. Intuitively, these augmentations can be seen as identity transformations on molecules (i.e., $x_i$ and $x_i'$ are two different strings representing the same underlying chemical). For instance, the canonical SMILES for methane is “C”, while the full version is “[CH4]” (carbon atom is connected to four hydrogen atoms). As in organic chemistry, a carbon atom C is implied to be connected to hydrogen atoms by default; hydrogen atoms H are usually omitted for brevity.

Overall, our AMORE framework can be briefly summarized as follows:

1. Take a set $X = (x_1, x_2,\dots ,x_n)$ consisting of *n* molecular representations;
2. Apply a transformation *f* to obtain a set of augmented molecular representations $X' = (x_1', x_2',\dots ,x_n')$, where $x_i' = f(x_i)$. The only constraint introduced for *f* is that it should not change an underlying chemical. We execute all augmentations through RDKit, a widely recognized methodology within the chemistry domain [24]. As in this work, we focus on textual molecular representations, we can think of $x_i$ and $x_i'$ as being **synonyms**.
3. For each $x_i \in X$ and $x_j' \in X'$ obtain their vectorized representations $e(x_i)$ and $e(x_j')$, respectively.
4. Evaluate the vectorized representations in a retrieval task: given an embedding $e(x_i)$, a model should be able to retrieve an embedding $e(x_i')$ of augmented $x_i'$.

The augmentation vectors are in the same embedding space, allowing distance measurement between original and augmented molecules. The better a model performs on the AMORE retrieval task, the more robust it is to the transformation *f*, suggesting that the model **recognizes** *f* is a **mapping between synonymous** representations.

***Datasets***

Our evaluation strategy relies on two popular datasets: (i) a ChEBI-20 test set [27] and (ii) a subset from the QM9 [28, 29] (further called **Isomers**), consisting of different molecules which are isomers of C9H12N2O. We select these datasets for the following reasons:

1. Utilizing the ChEBI-20 test set, which comprises approximately 3k molecule-description pairs, allows for comparisons with metrics such as ROUGE [19] and METEOR [20] in the molecule captioning task. The ChEBI-20 train set was used to train cross-domain ChemLMs. Hence, we follow the recent papers [11, 12], which use ChEBI-20 for benchmarking on molecule captioning tasks.
2. The ChEBI-20 dataset comprises molecular structures that translate into SMILES strings of varying lengths. This diversity in sequence length and symbol sets could potentially impact the mean characteristics of accurately identified results.
3. Furthermore, some molecules in the ChEBI-20 dataset may not be suitable for augmentation using our proposed methods. For instance, cycle renumbering relies on aromatic hydrocarbons, which are absent in non-organic compounds. This limitation may affect the comprehensiveness of our evaluation.

Due to these reasons, it is essential to complement the evaluation with datasets that mitigate these weaknesses. Therefore, we have selected molecules from the QM9 dataset presented in the PubChem database [30]. There are 3300 and 918 molecules in the ChEBI-20 test set and the Isomers set, respectively.

***Models***

For our experiments, we adopted various Transformer-based [1] molecular representation models. All models are publicly available at HuggingFace.

*Text+Chem T5* [12] is a multi-task, cross-domain language model that unifies natural language and chemical representations. It employs a shared T5 [14] encoder-decoder to learn from aligned text-SMILES pairs. For our experiments, we adopted two Text+Chem T5 *base*-sized models: (i) *Text+Chem T5-standard*, which is pre-trained on these 11.5M samples, and (ii) *Text+Chem T5-augm* which is pre-trained on an augmented version of this corpus that consists of 33.5M paired samples.

*MolT5* [11] is a self-supervised learning framework for jointly training a model on molecule captioning and text-based molecule generation tasks. The model employs a multi-task pre-training pipeline [14] to learn from 100 M SMILES strings from the ZINC-15 database [7] and natural language texts from the C4 [14] corpus.

*PubChemDeBERTa* [31] adopts DeBERTa V3 [32] encoder to learn molecular representations on PubChem [33] via the replaced token detection pre-training task. The model simultaneously adopts a Siamese neural network architecture to learn from biological assays, molecular fingerprints, and textual features (such as a molecule’s description and title). The authors released two versions of the pre-trained model: (i) a base one (ii) and an augmented one, which was trained on augmented textual descriptions. In our work, we experimented with the augmented version as it achieved higher perplexity on a test set [31].

*ChemBERT-ChEMBL* is a BERT-based [15] model pre-trained on 1.7M molecules in SMILES format from the ChemBL [34] database via the masked-language modeling (MLM) objective.

*ChemBERTa* [3] is a RoBERTa-based [35] molecular representation model which is pre-trained on 100K SMILES strings from the ZINC [7] benchmark via the MLM objective.

*BARTSmiles* [2] is a BART-like [36] sequence-to-sequence molecular representation model pre-trained on 1.7B SMILES samples from the Zinc20 [37] chemical database.

*ZINC-GPT* is a GPT-like [38] autoregressive language model trained on 480K SMILES strings from the ZINC [7] database.

*ZINC-RoBERTa* is a RoBERTa-based [35] molecular representation model which is pre-trained on 480K SMILES strings from the ZINC [7] database via the MLM objective.

*SciFive* [39] is a uni-modal textual T5-based model pre-trained on the union of general-domain C4 corpus and 32 M abstracts from the PubMed database.^2^ We adopt the model for our experiments to investigate if special chemical LMs are needed or if simple training of a universal LM with both textual and chemical modalities is enough for chemistry-related tasks.

*nach0* [13] is a self-supervised learning framework for jointly training a model on molecule captioning and text-based molecule generation tasks. The model employs a multi-task pre-training pipeline [14] to learn from 100 M SMILES strings from the ZINC-15 database [7] and natural language texts from the C4 [14] corpus.

**Table 1:** Domain and parameter count for models used in this study. “Chem” and “Text” are uni-modal chemical and textual models. “Cross” stands for cross-domain (bi-modal) language and chemistry LMs

Model	Domain	# Params
Text+Chem T5-standard	Cross	220 M
Text+Chem T5-augm	Cross	220 M
MolT5-base	Cross	220 M
MolT5-large	Cross	770 M
SciFive	Text	220 M
PubChemDeBERTa	Chem	86 M
ChemBERT-ChEMBL	Chem	6 M
ChemBERTa	Chem	125 M
BARTSmiles	Chem	400 M
ZINC-RoBERTa	Chem	102 M
nach0	Chem	220 M
ZINC-GPT	Chem	87 M

***Encoder-only*** A common approach is to train BERT-based encoders on unlabeled SMILES using objectives like Masked Language Modeling. We evaluate: (i) *PubChemDeBERTa* [31], (ii) *ChemBERT-ChEMBL* [40], (iii) *ChemBERTa* [3], and (iv) *ZINC-RoBERTa* that are pre-trained on SMILES from various chemical databases, namely, PubChem [33] and ZINC [7]. Some models, e.g., ChemBERT-ChEMBL and ChemBERTa, are known to be trained with augmented data.

***Encoder-decoder*** We focus on two recent T5-based [14] for text-related chemical tasks: (i) *Text+Chem T5* [12], (ii) *MolT5* [11] and (iii) *nach0* [13]. We utilize base and large versions of *MolT5* and two base-sized versions of *Text+Chem T5*. Additionally, we employed a biomedical LM *SciFive* [39], a uni-modal textual T5-based model pre-trained on the general-domain C4 corpus and PubMed database.

***Decoder-only*** As a decoder-only model, we adopt *ZINC-GPT* [41], a GPT-like [38] autoregressive language model trained on 480K SMILES from the ZINC database.

## Experimental results

**Table 2:** Top-1/Top-5 accuracy (%) and Mean Reciprocal Rank (MRR) of ChemLMs for matching of distributed representations of molecules with their augmentations on the ChEBI-20 dataset

Model	Canon			Hydro			Kekul			Cycle		
	Acc@1	Acc@5	MRR	Acc@1	Acc@5	MRR	Acc@1	Acc@5	MRR	Acc@1	Acc@5	MRR
Cross-modal models												
Text+Chem T5-standard	63.03	82.76	72.4	5.46	10.85	8.6	76.76	92.03	83.8	96.7	99.82	98.2
Text+Chem T5-augm	60.64	82.79	70.9	5.61	12.64	7.1	77.09	92.06	84.4	97.18	99.7	98.3
MolT5-base	55.64	59.79	50.9	5.97	7.27	5.5	62.76	80.52	70.9	90.94	97.18	93.8
MolT5-large	46.94	63.58	54.7	2.36	5.06	4.1	59.7	75.84	67.2	98.21	100	99.1
Unimodal models												
BARTSmiles	25.76	38.09	31.8	1.21	2.15	2.2	39.03	54.97	46.9	61.67	71.24	66.2
ZINC-GPT	23.85	33.85	28.8	0.85	1.64	1.5	35.09	48.45	41.7	75.3	85.03	80.1
SciFive	29.73	44.94	39.9	2.58	4.64	2.9	48.21	68.15	62.4	98.48	100	99.2
PubChemDeBERTa	32.79	48.09	40.3	2.15	4.33	3.6	53.55	73.15	62.9	96.39	99.45	97.9
ChemBERT-ChEMBL	26.06	37.79	32.2	1.73	3.3	2.8	37.7	54.91	46.1	79.55	87.03	83.2
ChemBERTa	26.61	40.12	33.3	1.09	2.3	2.1	44.18	65.42	54.1	92.58	98.42	95.3
ZINC-RoBERTa	23.33	33.61	33.2	0.97	2.39	1.7	33.09	46.97	45.5	90.61	97.48	69.2
nach0	45.27	61.42	53.25	2.72	5.27	4.68	72.03	86.67	78.87	92.39	98.69	95.09

**Table 3:** Top-1/Top-5 accuracy (%) and Mean Reciprocal Rank (MRR) of ChemLMs for matching of distributed representations of molecules with their augmentations on the Isomers dataset

Model	Canon			Hydro			Kekul			Cycle		
	Acc@1	Acc@5	MRR	Acc@1	Acc@5	MRR	Acc@1	Acc@5	MRR	Acc@1	Acc@5	MRR
Cross-modal models												
Text+Chem T5-standard	36.93	59.8	72.41	0.65	2.94	8.57	42.92	66.34	83.78	80.94	98.58	98.18
Text+Chem T5-augm	39	63.62	70.89	0.65	5.12	7.11	45.21	70.7	84.39	80.94	98.58	98.35
MolT5-base	29.96	44.55	37.62	0.54	3.16	2.65	36.17	51.96	44.32	76.36	92.37	83.52
MolT5-large	29.41	42.81	37.45	1.53	6.75	3.16	35.29	49.13	43.41	81.7	98.15	90.72
Unimodal models												
BARTSmiles	27.89	42.05	31.76	0	0.87	1.11	31.81	48.58	37.38	41.83	44.77	42.43
ZINC-GPT	24.18	36.17	32.03	0.44	1.31	0.97	27.45	43.03	37.69	55.12	68.52	64.41
SciFive	22	33.44	39.95	0	1.2	2.97	24.62	37.8	62.41	93.14	98.04	99.22
PubChemDeBERTa	26.69	38.13	31.96	0.22	0.65	0.99	31.59	44.88	37.8	87.36	94.99	90.82
ChemBERT-ChEMBL	23.64	34.86	31.52	0.98	3.38	1.34	27.12	39.54	36.23	37.15	39.76	65.99
ChemBERTa	25.93	36.6	31.69	0.65	2.94	1.74	29.3	41.72	36.46	50.98	60.13	80.49
ZINC-RoBERTa	28.76	42.27	36.48	0.65	1.85	1.33	33.12	49.35	42.61	50.76	56.86	84.64
nach0	33.66	54.24	43.88	0.65	2.94	2.80	39.54	62.20	50.64	61.77	79.96	70.46

**Table 4:** Top-1/Top-5 accuracy (%) and Mean Reciprocal Rank (MRR) of ChemLMs for matching of distributed representations of molecules with their random augmentations on the ChEBI-20 and Isomers datasets

Model	Random ChEBI-20			Random isomers		
	Acc@1	Acc@5	MRR	Acc@1	Acc@5	MRR
Cross-modal models						
Text+Chem T5-standard	46.94	74.18	59.33	15.80	38.13	27.17
Text+Chem T5-augm	51.21	76.72	65.84	18.63	44.12	31.58
MolT5-base	28.82	51.06	39.50	11.44	22.66	18.01
MolT5-large	23.18	40.73	31.96	7.84	16.67	13.01
Unimodal models						
BARTSmiles	15.55	30.24	23.05	7.29	12.53	10.72
ZINC-GPT	6.64	12.67	10.24	6.54	11.00	9.51
SciFive	22.64	40.73	31.40	6.65	15.36	11.83
PubChemDeBERTa	22.27	39.21	30.47	7.52	15.36	11.68
ChemBERT-ChEMBL	22.79	41.27	31.94	8.28	19.50	14.58
ChemBERTa	14.58	28.81	21.68	8.06	16.23	13.18
ZINC-RoBERTa	15.88	28.88	22.84	9.48	20.15	15.43
nach0	24.49	44.64	34.22	14.38	33.55	24.56

### Molecule-augmentation retrieval

Given an original SMILES $x_i$, we rank all augmented representations $x_j'$ in terms of similarity between pooled representations $e(x_i)$ and $e(x_j')$ obtained from a chemical LM. We assume that if a model retrieves an augmented $x_i'$ of a higher rank given $x_i$, it is robust to the selected augmentation and is aware that the given augmentation is an identity transformation of the set of molecules. We use mean-pooled embeddings from Transformer layers as representations of SMILES.

Our results for matching distributed representations of molecules with their augmentations on ChEBI-20 and Isomers datasets are presented in Tables 2, 3, and 4. Higher top-1/top-5 accuracy and MRR indicate a model can recognize that varying SMILES representations correspond to the same molecule, i.e., robust to that type of augmentation.

***Chemical LMs are still not robust to SMILES augmentations***

The existing ChemLMs struggle to retrieve augmented SMILES for non-augmented ones indicating that they are unable to recognize synonymous SMILES variations. We provide experiments results in Tab. 5. We evaluated three recent models that show high scores on the main ChEBI-20 task on augmented ChEBI-20 datasets. The nach0 model shows the best performance on the canonicalised version of dataset. We assume that this effect is caused by specificity of pretraining and finetuning datasets: the results allow to propose that the main part of training dataset’s SMILES was converted to the canonical form. This model was not specially finetuned with ChEBI-20 testset, that caused low values of textual metrics on ChEBI-20 molecule captioning task.

The finding suggests that pre-training on SMILES leads to memorization rather than an actual understanding of chemistry and results in a poor generalization. No model performs best on all augmentations and datasets, but retrieval is higher on the less complex ChEBI-20 dataset, possibly due to the transformation of short and non-aromatic molecules by our augmentations being less frequent.

***Robustness to different types of augmentations varies significantly***

For all ChemLMs, augmentation ordering concerning retrieval accuracy remains consistent: the most challenging augmentation is explicit hydrogen addition, then transforming into RDKit canonical, kekulization, and cycle renumbering. Encoder-only PubChemDeBERTa, ChemBERTa, and ZINC-RoBERTa models are not far behind T5 models for cycle renumbering augmentation on ChEBI-20. Surprisingly, retrieval accuracy for hydrogen addition augmentation is extremely low for all models. On Isomers, all models have failed to surpass 1% accuracy. We believe that poor performance on hydrogen addition is caused by its absence in pre-training data of these models: hydrogen is always omitted whenever possible.

The model that shows unusual results, nach0, was trained on the MolInstructions data for the molecule captioning task. Due to novelty of captioning format, ROUGE and METEOR metrics are lower than other models metrics, still the model generate natural descriptions that contain plausible information and characteristics (for examples, see Supplementary materials).

**Table 5:** Detailed evaluation results of ChemLMs for the ChEBI-20 test set: top-1 accuracy (Acc@1, %) for matching of distributed representations of molecules with their augmentations and ***ROUGE2*** and ***METEOR*** for matching of textual outputs of LMs with gold descriptions (molecule captioning task)

Augmentation $\longrightarrow$	canon			hydro		
Metrics	Acc@1	***ROUGE2***	***METEOR***	Acc@1	***ROUGE2***	***METEOR***
Text+Chem T5-standard	63.03	0.381	0.515	5.46	0.187	0.314
Text+Chem T5-augm	60.64	0.377	0.514	5.61	0.201	0.336
MolT5-base	42.88	0.315	0.450	2.36	0.199	0.329
MolT5-large	46.94	0.390	0.532	2.7	0.174	0.317
nach0-base	45.27	0.201	0.234	2.72	0.134	0.170

Augmentation $\longrightarrow$	kekul			cycles		
Metrics	Acc@1	***ROUGE2***	***METEOR***	Acc@1	***ROUGE2***	***METEOR***
Text+Chem T5-standard	76.76	0.413	0.574	96.7	0.483	0.600
Text+Chem T5-augm	77.09	0.410	0.546	97.18	0.458	0.581
MolT5-base	62.76	0.333	0.475	90.94	0.417	0.540
MolT5-large	59.7	0.405	0.546	98.21	0.477	0.603
nach0-base	72.03	0.189	0.219	92.39	0.171	0.204

Augmentation $\longrightarrow$	random					
Metrics	Acc@1	***ROUGE2***	***METEOR***			
Text+Chem T5-standard	46.94	0.357	0.499			
Text+Chem T5-augm	51.21	0.370	0.507			
MolT5-base	28.82	0.277	0.417			
MolT5-large	26.18	0.338	0.490			
nach0-base	24.49	0.167	0.196			

Here, canon refers to RDKit canonicalization, hydro to Hydrogen explicit addition, kekul to Kekulization, and cycles to cycle renumbering. The metrics ***ROUGE2*** and ***METEOR***, in blod italic, are the captioning metrics

***Chemical LMs benefit from cross-modality***

For four augmentations except for cycle renumbering, cross-modal models (MolT5 and Text+Chem T5 variations) pre-trained on textual and chemical tasks yield higher retrieval accuracy consistently. The Text+Chem T5 standard and Text+Chem T5-augm scores are, in most cases, higher than the scores of other models. Interestingly, SciFive is the most robust to cycle renumbering on both datasets, even though it is pre-trained only on texts with no SMILES. The top-1 accuracy obtained matches the top-5 accuracy. The highest absolute top-5 accuracy gain is observed for encoder-decoder cross-modal Text+Chem T5 models.

### AMORE and captioning quality

***Captioning quality is consistent with AMORE*** From Table 5, the most significant drop in ROUGE and METEOR is observed for the *hydrogen addition* augmentation, which is consistent with our proposed AMORE metric. While ROUGE and METEOR require labor-intensive labeled datasets for evaluation, our proposed embedding distance-based AMORE framework supports zero-shot evaluation and only requires a set of SMILES strings. Though the correlation between Acc@1 and ROUGE/METEOR is not straight forward, we found that the differences between caption metrics for original and augmented SMILES strings correlate with the distributional metrics from the AMORE (for example, the Spearman correlation for Acc@1 is greater than 0.7 with p-value=0.003). This means that even in the case of absence of labeled datasets, the AMORE framework allows to predict, how the augmentations will affect captioning metrics.

**Fig. 2:**
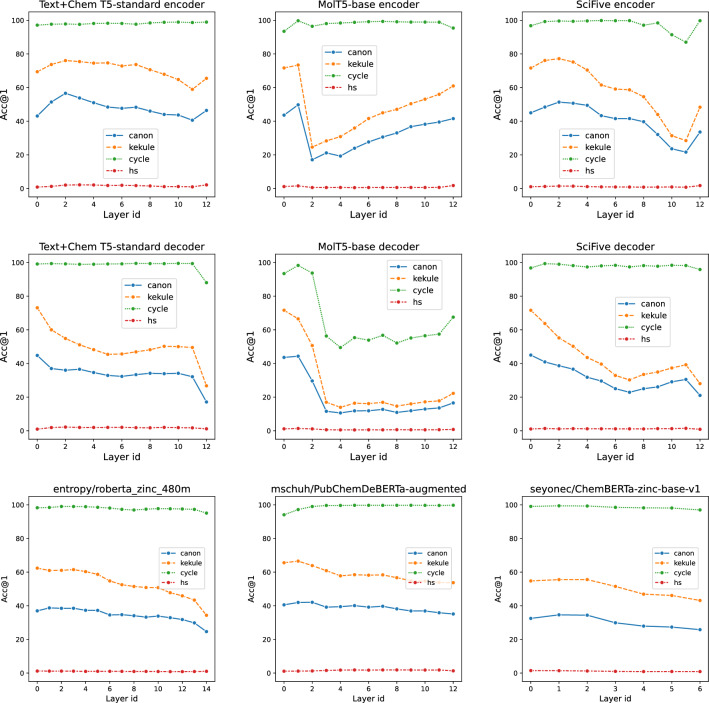
Top-1 retrieval accuracy (Acc@1) on ChEBI-20 dataset calculated for hidden representations for different layers of encoder-decoder chemical LMs. The 0-th layer is the initial token embeddings (embedding layer) before any Transformer layers. The first row presents the results for encoders; the second row stands for decoders

***Representation robustness correlates across different augmentations*** The flexibility of our framework allows us to take hidden representations from an arbitrary intermediate layer of a ChemLM. We explored how the retrieval-based top-1 accuracy changes over different Transformer layers. Figure 2 presents the layer-wise AMORE metric for T5 and BERT-like models. An interesting finding is that layer-wise retrieval quality strongly correlates across varying augmentation types. For instance, Text+Chem T5-standard faces a significant top-1 accuracy drop on the 12th decoder layer for three of four augmentation types simultaneously. The same stands for SciFive’s decoder. For MolT5’s encoder and decoder, a notable performance drop is observed for the 3rd layer. BERT-like models show a tendency for a gradual decrease in metrics. However, layer dynamics is not consistent across different Chem LMs.

***How does the type of metric influence on AMORE?***

We compared 4 different approaches for distance calculation: default $L_2$, *cosine*, *Inner Distance* and *HNSW*([42]). The results are summarized in Table 6. For all the augmentations except Explicit Hydrogen, the standard L2 approach works the best. In the case of the explicit Hydrogen, cosine and HNSW allow one to arrange the embeddings better than the default $L_2$ metric.

**Table 6:** Top-1/Top-5 accuracy (%) of Text+Chem T5-augm model for matching of distributed representations of molecules with their random augmentations on the ChEBI-20 dataset for different metric choices

Metric type	Canon		Hydro		Kekul		Cycle		Random	
	Acc@1	Acc@5	Acc@1	Acc@5	Acc@1	Acc@5	Acc@1	Acc@5	Acc@1	Acc@5
$L_2$	60.64	80.79	5.61	12.64	77.09	92.06	97.18	99.70	51.21	76.72
Cosine	57.82	78.33	8.97	21.85	76.27	91.88	97.24	99.67	47.94	72.79
Inner distance	18.19	40.46	5.18	15.97	27.79	57.55	47.36	78.91	13.76	34.39
HNSW	57.85	78.24	9.00	21.70	76.27	91.88	97.24	99.67	47.79	72.79

***Levenshtein: discrepancy between different types of augmentations*** To further understand the root causes of ChemLM’s performance degradation on augmented test sets, we explored the dependency between molecule captioning quality on ChEBI-20 and simpler SMILES string properties. In particular, for each augmented test set, we measure average string length and the Levenshtein distance between the original SMILES and an augmented one. For each pair of original and augmented SMILES, we define Levenshtein ratio as the ratio between their Levenshtein distance and the length of the original SMILES string. Additionally, we include the Spearman’s correlations between the target metrics, such as ROUGE1 and METEOR, and the Levenshtein ratio for MolT5 model. The results are shown in Tables 7 and 8. While high string length (Levenshtein ratio for hydrogen augmentation is three times larger than for canonicalization, kekulization and randomized order cases) could partially explain poor generalization on hydrogen addition augmentation, low correlation values between the target metrics and Levenshtein ratio indicates that string variation is not the only challenge. A deeper insight into generalization limitations on augmented data requires a future work.

**Table 7:** Levenshtein ratio for different types of augmentations, raw strings

Augmentation type	SMILES length (mean/std)	Levenshtein ratio with the original string (mean/std)	Correlation between Levenshtein ratio and ROUGE1	Correlation between Levenshtein ratio and METEOR
No augmentation	78.96 (80.29)	0	–	–
Canon	74.71 (78.06)	0.47 (0.22)	$-$ 0.33	$-$ 0.34
Hydro	153.36 (134.42)	1.49 (0.54)	$-$ 0.05	$-$ 0.09
Cycles	78.97 (80.29)	0.04 (0.04)	$-$ 0.34	$-$ 0.33
Kekul	76.98 (78.18)	0.40 (0.20)	$-$ 0.24	$-$ 0.22

**Table 8:** Levenshtein ratio for different types of augmentations, tokenized strings

Augmentation type	Tokenized SMILES length (mean/std)	Levenshtein ratio with the original representation (mean/std)
no augmentation	61.68 (63.54)	0
canon	55.89 (58.90)	0.53 (0.23)
hydro	134.34 (115.88)	2.23 (2.92)
cycles	61.93 (63.65)	0.04 (0.04)
kekul	58.95 (59.31)	0.46 (0.22)
random	60.56 (61.17)	0.65 (0.35)

*Deeper dive into the Explicit Hydrogen augmentation*

**Fig. 3:**
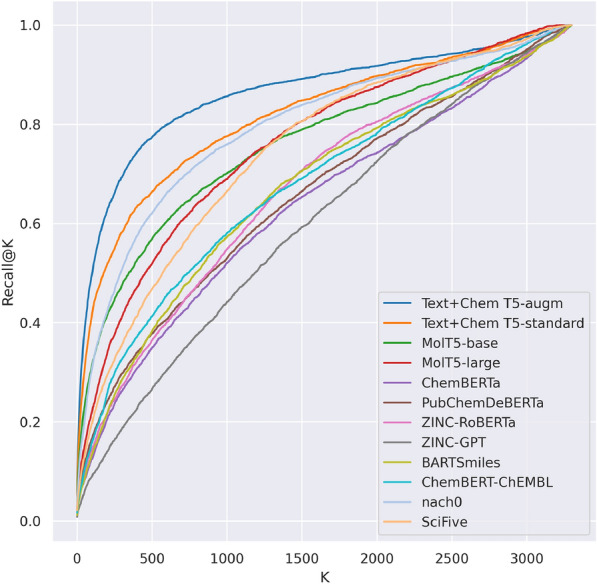
AMORE Recall@K curves for the Explicit Hydrogen augmentation on ChEBI-20 dataset

All the models show significant drops of quality for the Hydrogen addition augmentation. We suspect that such behavior is partly caused by the token distribution shift; for instance, while “CH”, “CO”, “NH” tokens are rare in the case of the original ChEBI-20 SMILES strings, they become frequent after the augmentation. Additionally, we measured Recall@K scores in terms of AMORE to get more information concerning augmented SMILES embeddings and plotted them as Recall curves Fig. 3. In general, we see that Recall curves behave in a way similar to Acc@(1,5).

### AMORE and additional downstream tasks

**Fig. 4:**
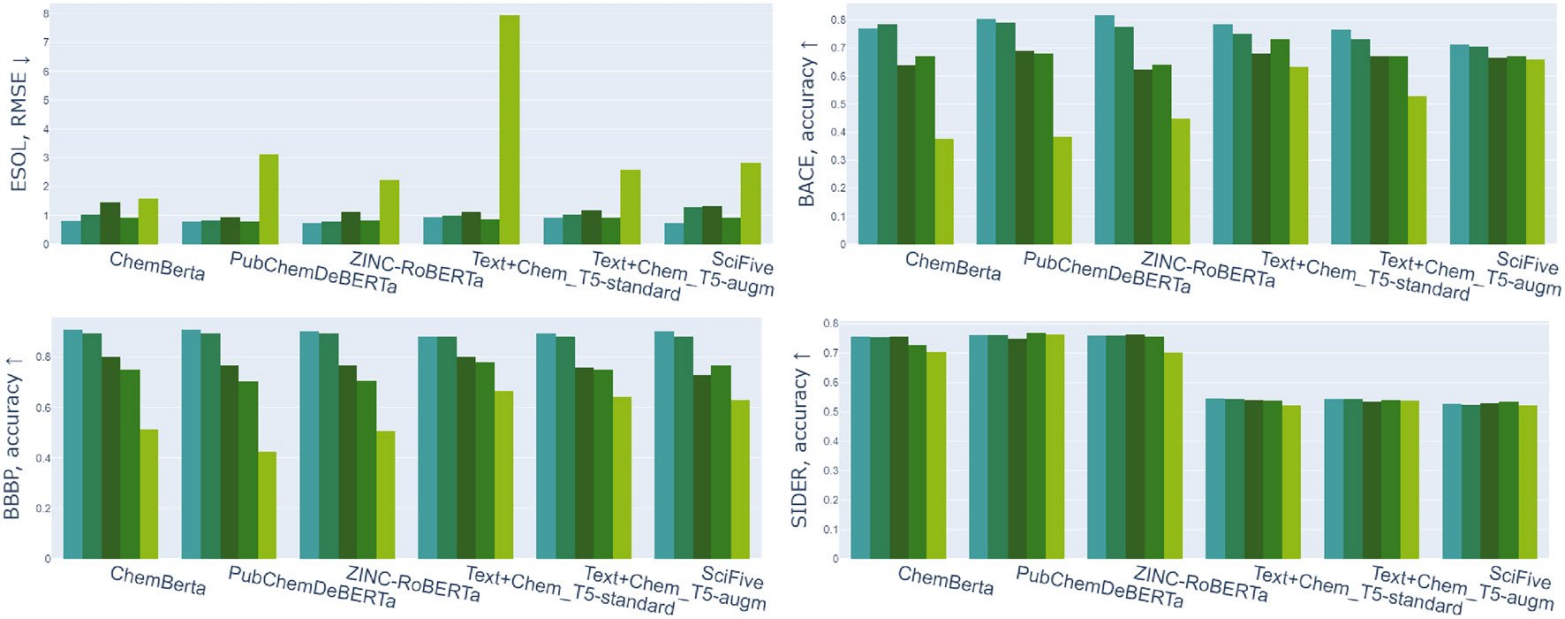
Performance on original and augmented MoleculeNet test sets, showing the impact of different data augmentation techniques on model performance across regression (ESOL), binary classification (BBBP, BACE), and multilabel classification tasks (SIDER). Bars represent left to right: identical, canonical, kekulized, cycle and explicit hydrogen augmentations

In order to additionally explore the impact of the proposed augmentations, we utilize the MoleculeNet [43] benchmark with chemistry tasks. The MoleculeNet benchmark is the established standard in the research community to assess and compare the performance of models on various molecular property prediction tasks, spanning topics from quantum mechanics to physiology. We consider 9 tasks from it: three regression tasks (Lipophilicity, ESOL, FreeSolv), 3 binary classification tasks (HIV, BBBP, BBPA), and 3 multilabel classification tasks (Tox21, ToxCast, SIDER).The results are presented in Fig. 4.

*Augmented SMILES lead to degraded performance on chemical tasks*

Experiments showed metrics generally degrade on augmented MoleculeNet test sets (Fig. 4). For example, RMSE on the ESOL regression task increased from 0.87 to 7.93 with hydrogen addition. However, not all augmentations had the same impact, with cyclic augmentations having a smaller effect (0.93–0.99 for Text+ChemT5-standard). The impact of augmentations was more distinct in binary classification (BACE). PubChemDeBerta accuracy dropped from 0.8 to 0.38 with hydrogen addition, with intermediate drops for other augmentations. The major part of the model’s accuracy range in binary classification (BBBP) is the following: original–cycle–Kekule–canon–hydrogen. For multilabel classification, BERT-based models (PubChemDeBerta, ChemBerta) outperformed T5-based models, suggesting the latter may not be well-suited for tasks with many classes.

*Chemical LM Ranking*

To explore how the ranking of ChemLMs on augmented test sets changes compared to non-augmented data, we conduct our experiments on nine datasets MoleculeNet datasets as follows. Each model is trained on the original train set provided in MoleculeNet and evaluated on both the original test set and four augmented test sets. Next, we rank all models with respect to their performance on a given test set type (either an original one or one of four augmented ones) using the Vote’n’Rank framework [44]. The framework is designed for ranking systems in multi-task benchmarks under the principles of the social choice theory [45]. We follow recommendations from [44] and use Copeland rule to select the system that beats all the others in pairwise comparison. Copeland chooses the system that dominates the others in more cases and is dominated by the least.

The results are presented in Table 9. Overall, all augmentations except for hydrogen addition do not seem to significantly alter the original ranking. For instance, Zinc-RoBERTa and PubChemDeBERTa achieved rank 1 and 2, respectively, on four of five test sets. Similarly, MolT5-base placed last on all augmentations except for hydrogen addition. It seems that encoder-decoder architectures are more stable to hydrogen addition on downstream tasks than encoder-only architectures as 4 of top 5 places are achieved by MolT5-large, MolT5-base, Text+Chem T5-augm, and SciFive.

**Table 9:** ChemLM rankings with respect to Vote’n’Rank framework’s Copeland score calculated on 9 downstream tasks from the MoleculeNet benchmark for different augmentation types

Rank	Test set				
	Original	Canon	Hydro	Kekul	Cycles
1	$\ddagger$	$\ddagger$	$\diamondsuit$	$\ddagger$	$\ddagger$
2	$\dagger$	$\dagger$	♥	$\dagger$	$\dagger$
3	$\Box$	♦	$\spadesuit$	♦	$\Box$
4	$\spadesuit$	$\Box$	$\dagger$	$\Box$	$\spadesuit$
5	♦	$\spadesuit$	$\triangle$	$\spadesuit$	♦
6	$\clubsuit$	$\diamondsuit$	$\clubsuit$	$\clubsuit$	$\clubsuit$
7	$\triangle$	$\triangle$	$\ddagger$	$\diamondsuit$	$\triangle$
8	$\diamondsuit$	$\clubsuit$	$\Box$	$\triangle$	$\diamondsuit$
9	♥	♥	♦	♥	♥

Here, canon refers to RDKit canonicalization, hydro to Hydrogen explicit addition, kekul to Kekulization, and cycles to cycle renumbering. **Models:** $\ddagger$=ZINC-RoBERTa, $\dagger$=PubChemDeBERTa, $\Box$=ChemBerta, $\clubsuit$=Text+Chem T5-augm, ♦=Text+Chem T5-standard, $\clubsuit$=Text+Chem T5-augm, $\triangle$=SciFive, $\diamondsuit$=MolT5-large, ♥=MolT5-base

## Discussion

In this paper, we offer a general framework for analyzing knowledge awareness of modern LMs in the chemical domain. Although we rely on the L2 distance as a similarity distance throughout all our experiments, an arbitrary embedding similarity measure can be employed. Similarly, possible augmentation types are not limited to the ones considered in our research and can be extended. This flexibility might open up new avenues for the interpretation and analysis for LMs in the chemical domain.

Our experiments have shed light on the research question formulated in the Introduction and revealed a few critical limitations of the existing LMs in chemistry-related tasks. First, the embedding space of chemical LMs is not robust even to simple augmentations of SMILES strings known as identity transformations of molecules in chemistry. Although robustness to these augmentations can vary across different model layers, no intermediate layer would be stable to SMILES augmentations. Second, the performance of chemical LMs in downstream tasks, such as molecule captioning, can be significantly limited when an out-of-distribution (OOD) input. These two findings demonstrate that **the existing chemical LMs have problems distinguishing the same molecules in different representations** during NLP-inspired pre-training procedures. They overfit on a specific format of input molecular string representations rather than truly gain an understanding of molecules. Finally, cross-modal chemical LMs tend to be more robust to OOD input samples, highlighting the importance of further developing multimodal models for chemistry and NLP. Meanwhile, the metrics for the isomers dataset are lower and show minimal differences across models, probably attributed to the structure of the data set comprising isomeric aromatic compounds with identical molecular formulas and atom counts.

The key idea is that chemical models must accurately translate augmented SMILES into molecular structures. Without fully understanding the syntax of SMILES and distinguishing same-structure SMILES, ChemLMs remain vulnerable to real-world data perturbations. This analysis aims to inform revisions to the established pipeline for learning chemical representations from NLP.

The proposed framework may serve as a regularization tool to enhance the robustness of new models. For instance, one may employ metric learning techniques ([46]) to encourage trained models to embed the variants of a given SMILES close to each other.

## Conclusion

In this paper, we introduce AMORE, a novel method (Fig. 1) based on embedding distance and SMILES augmentation to explore and evaluate the model’s representations of a chemical substance and its ability to recognize molecule structures in SMILES string representations. Using this method, we assessed the most popular chemical LMs for several benchmarks (ChEBI-20, QM9). We propose to use an isomeric subset of the QM9 dataset, which is novel to this task.

Though the first attempts to study the impact of chemical augmentations on Text+Chem T5 and MolT5 for molecule captioning exist, this is limited to cross-domain generative architectures requiring NLP tokens, constraining the number of suitable models for evaluation. The key novelty of our paper lies in the proposed probing scheme. It is the first application of computation of distances between embeddings for benchmarking chemical LLMs. As a result, our AMORE framework drastically extends this scope for evaluating and comparing models in domain-specific diverse architectures, including encoder-only versus generative models, as well as uni-modal LMs (with molecule atom tokens only) versus cross-modal models (atom + NLP tokens). It is important to emphasize that our method exploits unique specifics of the chemical domain. In contrast with typical NLP tasks, our augmentations lead to the creation of total synonyms of a molecule, which are absent in general words of natural language.

Our framework opens avenues for future research, ranging from understanding the functionality of molecule SMILES representations in LMs to addressing weaknesses in chemical tasks and enhancing efficiency.

## Additional file

Supplementary file 1.

## Data Availability

All purposed data, methodology, and code are available at: ChemistryLLMs Github (2024) Code and data of AMORE framework. https://github.com/ChemistryLLMs/AMORE Accessed 22 Feb 2025.

## Footnotes

1 AMORE is available at https://github.com/ChemistryLLMs/AMORE

2 https://pubmed.ncbi.nlm.nih.gov

## Declarations

### Limitations

First, we evaluated modes that are publicly available at HuggingFace (HF). We note that there are other popular models such as Chemformer (https://github.com/MolecularAI/Chemformer), Molformer (https://github.com/IBM/molformer) and T5Chem (https://github.com/HelloJocelynLu/t5chem), which we failed to plug as HF checkpoints. Second, the evaluated models primarily focus on the sequence format of molecules, but it is important to consider in future other formats, such as 3D structures, which also hold significant importance. Third, we emphasize that the evaluated models were developed for research purposes and may contain unintended biases, and any molecules generated by them should undergo thorough evaluation through standard clinical testing. Furthermore, SELFIES [47] and other molecule naming systems are also widespread in the chemical field. In our research, we have focused on SMILES due to its popularity, but the augmentations of other systems are yet to be explored.
