# Supplementary material for: Measuring Chemical LLM robustness to molecular representations: a SMILES variation-based framework
Source: J Cheminform. 2025 Oct 30;17:164. doi: 10.1186/s13321-025-01079-0 (PMC12574305; doi:10.1186/s13321-025-01079-0)
Supplement: Supplementary file 1 [file 13321_2025_1079_MOESM1_ESM.pdf]

# Supplementary material

## 1 Qualitative Analysis of generated molecule description

As written in the section below, machine translation metrics can't hangle complexity of domain-specific words. In order to support this, we perform quantitative analysis to specify the nature of errors. Qualitative analysis allows for a detailed examination of mistakes, aiding in diagnosing specific issues, which is vital for model improvement. We provide several examples of model-generated descriptions and analyze their alignment with the actual properties of the corresponding molecules. These descriptions were created using both original and augmented datasets through the Text+Chem T5-augm (Christofidellis et al, 2023) and nach0 (Livne et al, 2024) models.

The selected molecule for this analysis is duloxetine hydrochloride, taken from the test section of the CHEBI-20 dataset (Edwards et al, 2021). An output example from the Text+Chem T5-augm model, specifically for duloxetine hydrochloride, can be found in Table 1. Additionally, outputs from the nach0 model related to duloxetine hydrochloride predictions are shown in Table 2.

The predictions regarding the substances and reactions generated by the models proved inaccurate across all datasets, including both augmented and original datasets. Each model's output comprised a blend of correct and incorrect information.

Cycle Augmentation: The model accurately identified duloxetine hydrochloride's role as an antidepressant and serotonin uptake inhibitor and correctly outlined its precursor reaction with hydrochloric acid, including the appropriate molar ratio. However, it mistakenly proposed irinotecan as a precursor and fails to clarify the presence of atomic sulfur in the resultant hydrochloride.

Original Test: The model posited that duloxetine hydrochloride results from a reaction between 1-2-(1-benzothiophen-3-yl)ethoxy-4-(methylamino)butanal and hydrochloric acid. While it correctly identified the substance class and medical role, the precursor appears to be nonexistent, as it is not listed on PubChem nor in any relevant literature, despite components resembling it being present in the training set.

| Probing Test | Input | Output |
|---|---|---|
| original | `CNCCC(C1=CC=CS1)OC2=CC=CC3=CC=CC=C32.Cl` | The molecule is a **hydrochloride salt** resulting from the reaction of **1-[2-(1-benzothiophen-3-yl)ethoxy]-4-(methylamino)butanal** with **1 mol eq.** of **hydrogen chloride**. It has a role as an **antidepressant** and a **serotonin uptake inhibitor**. It contains a **1-[2-(1-benzothiophen-3-yl)ethoxy]-4-(methylamino)butanal**. |
| canonical | `CNCCC(Oc1cccc2ccccc12)c1cccs1.Cl` | The molecule is a **hydrochloride** obtained by combining **equimolar** amounts of **cis-diltiazem** and **hydrogen chloride**. It has a role as an **antidepressant** and a **serotonin uptake inhibitor**. It contains a **cis-diltiazem(1+)**. |
| hydrogen | `[CH3][NH][CH2][CH2][CH]([O][c]1[cH][cH][cH][c]2[cH][cH][cH][cH][c]12)[c]1[cH][cH][cH][s]1.[ClH]` | The molecule is an **ammonium salt** resulting from the reaction of **equimolar** amounts of **scyllo-inositol** and **ammonia**. It contains a **scyllo-inositol(1+)**. It derives from a **hydride of a scyllo-inositol**. |
| kekulization | `CNCCC(OC1=CC=CC2=CC=CC=C12)C1=CC=CS1.Cl` | The molecule is a **hydrochloride** obtained by combining **N-methyltryptamine** with **one molar** equivalent of **hydrochloric acid**. It has a role as an **antidepressant** and a **serotonin uptake inhibitor**. It contains a **N-methyltryptamine(1+)**. |
| cycles | `CNCCC(C4=CC=CS4)OC2=CC=CC7=CC=CC=C72.Cl` | The molecule is a **hydrochloride** obtained by combining **irinotecan** with one molar equivalent of **hydrochloric acid**. It has a role as an **antidepressant** and a **serotonin uptake inhibitor**. It contains an **irinotecan(1+)**. |
| Pubchem | `CNCCC(C1=CC=CS1)OC2=CC=CC3=CC=CC=C32.Cl` | The molecule is a **duloxetine hydrochloride** in which the duloxetine moiety has S configuration. It has a role as an **antidepressant**. It contains a **(S)-duloxetine**. |

**Table 1** Examples of SMILES and descriptions generated by Chem+Text T5-base. We highlighted in bold the most important components of the description: precursors, role, and others.

Canonical Test: Similar to cycle augmentation, the model successfully recognized the roles and reactions of duloxetine hydrochloride. However, it erroneously identified cis-diltiazem as a precursor. This is problematic, as this calcium channel blocker should not react with gastric components to produce an antidepressant.

| **Probing Test** | **Input** | **Output** |
|---|---|---|
| original | `CNCCC(C1=CC=CS1) OC2=CC=CC3=CC=CC=C32.Cl` | The molecule is a **natural product** found in **Scytonema mirabile** with data available. |
| canonical | `CNCCC(Oc1cccc2ccccc12) c1cccs1.Cl` | The molecule is a **hydrochloride** obtained by combining **(R)-fenoldopa and hydrogen chloride**. A **reversible dual reuptake inhibitor** that has been shown to be as **effective and safe in patients with ADHD**. It has a role as an **atypical antipsychotic drug**, a **second generation antipsychotic**, an **antiemetic** and an **antidote to the actions of dopamine**. It contains a **(R)-fenoldopa(1+)**. It is an **enantiomer of a (S)-fenoldopa hydrochloride**. |
| hydrogen | `[CH3][NH][CH2][CH2][CH] ([O][c]1[cH][cH][cH] [c]2[cH][cH][cH][cH] [c]12)[c]1[cH][cH] [cH][s]1.[ClH]` | The molecule is a **hydroxamic acid** that is **hydrochloric acid** in which the **hydrogens at positions 1 and 2 have been replaced by hydrogens**. It is a **hydroxamic acid** and a **polycyclic aromatic hydrocarbon**. |
| kekulization | `CNCCC(OC1=CC=CC2=CC =CC=C12)C1=CC=CS1.Cl` | The molecule is a **hydrochloride salt** resulting from the reaction of **equimolar amounts of (S)- and (R)-epothilone A**. It has a role as a **histamine antagonist**, an **anti-allergic agent**, a **bronchodilator agent** and an **anti-arrhythmia drug**. It contains a **(S)-epothilone A(1+)**. |
| cycles | `CNCCC(C4=CC=CS4) OC2=CC=CC7=CC=CC=C72.Cl` | The molecule is the **hydrochloride salt of (R,R)-bisermol**. It has a role as a **plant metabolite**. It contains a **(R,R)-bisermol**. |
| Pubchem | `CNCCC(C1=CC=CS1) OC2=CC=CC3=CC=CC=C32.Cl` | The molecule is a **duloxetine hydrochloride** in which the duloxetine moiety has S configuration. It has a role as an **antidepressant**. It contains a **(S)-duloxetine**. |

**Table 2** Examples of SMILES and descriptions generated by nacn0 We highlighted in bold the most important components of the description: precursors, role, and others.

Hydrogen Augmentation: This augmentation yielded the least accurate predictions, with all substances and roles misidentified, demonstrating significant shortcomings.

Kekule: The model again accurately identified duloxetine hydrochloride's function and precursor reaction with hydrochloric acid. Yet, it mistakenly proposed N-methyltryptamine as a precursor, although this molecule does exist, it is not found in the training data.

Overall, the model struggled to deliver accurate predictions, particularly with augmented data. It correctly recognized duloxetine hydrochloride's role and the precursor reaction with hydrochloric acid across most augmentations, except for hydrogen addition. All predictions appeared to stem from a common high-frequency formula observed in the training data, often referencing hydrochlorides.

The predictions generated by the nach0 model (Livne et al, 2024) present several issues, although they differ in specifics from those of other models.

Cycle Augmentation: The model correctly identified duloxetine hydrochloride as a hydrochloride salt. However, it inaccurately labeled it as a plant metabolite, a claim not supported by patents or chemical resources. Furthermore, (R,R)-bisermol does not appear to exist in chemical databases.

Original Test: The model suggests that duloxetine hydrochloride is a natural product found in Scytonema mirabile, a type of photosynthetic cyanobacteria. This is erroneous because Scytonema mirabile does not utilize duloxetine as a metabolite, rendering this prediction false.

Canonical Test: While the model accurately classified duloxetine hydrochloride, it mistakenly predicted its precursors. Although (R)-fenoldopa seems to reference an existing drug (fendolopram), this connection is flawed. The model correctly identifies duloxetine as a dual reuptake inhibitor for serotonin and norepinephrine, indicating potential benefits for ADHD patients. Notably, duloxetine is not classified as an antipsychotic or antiemetic.

Hydrogen Augmentation: This model iteration failed to provide any role-specific information about the molecule. Additionally, it inaccurately described the molecular class, as duloxetine is a salt and not a hydroxamic acid. The phrase "hydrogens have been replaced by hydrogens" is grammatically correct but lacks coherence.

Kekule Test: The model adeptly identified duloxetine hydrochloride's role and precursor reaction with hydrochloric acid, including the correct molar ratio. However, it incorrectly suggested N-methyltryptamine as a precursor. While this molecule exists, it does not appear in the training data.

In summary, both models struggle to deliver accurate predictions in both augmented and non-augmented tests, even when pre-trained on different datasets. They consistently make similar errors, often relying on clichéd expressions like "The molecule is... It contains... It is a drug/plant metabolite." Such templates may yield high evaluation scores when they match original formulations, yet this does not guarantee factual accuracy, revealing a critical flaw in both models' predictive capabilities.

# References

Christofidellis D, Giannone G, Born J, et al (2023) Unifying molecular and textual representations via multi-task language modelling. In: Krause A, Brunskill E, Cho K, et al (eds) International Conference on Machine Learning, ICML 2023, 23-29 July 2023, Honolulu, Hawaii, USA, Proceedings of Machine Learning Research, vol 202. PMLR, pp 6140–6157, URL https://proceedings.mlr.press/v202/christofidellis23a.html

Edwards C, Zhai C, Ji H (2021) Text2Mol: Cross-modal molecule retrieval with natural language queries. In: Proceedings of the 2021 Conference on Empirical Methods in Natural Language Processing, pp 595–607, URL https://aclanthology.org/2021.emnlp-main.47/

Livne M, Miftahutdinov Z, Tutubalina E, et al (2024) nach0: multimodal natural and chemical languages foundation model. Chem Sci 15:8380–8389. https://doi.org/10.1039/D4SC00966E, URL http://dx.doi.org/10.1039/D4SC00966E
